# IL13Rα2 signaling in colorectal cancer

**DOI:** 10.18632/oncoscience.241

**Published:** 2015-09-12

**Authors:** Rubén A. Bartolomé, J. Ignacio Casal

**Affiliations:** Department of Cellular and Molecular Medicine, Centro de Investigaciones Biológicas (CIB-CSIC), Madrid, Spain

**Keywords:** IL13Rα2, IL13, FAM120A, cell signaling, colon cancer

IL13 receptor α2 (IL13Rα2) is a high-affinity IL13 receptor overexpressed in a variety of human cancer types. In many of these cancers (glioblastoma, head and neck squamous cell carcinoma and ovarian cancer) the expression of this receptor is associated with poor prognosis [1–3]. In colorectal cancer cells, IL13 signaling through its receptor IL13Rα2 plays a critical role in cell adhesion, invasion and liver metastasis. Moreover, overexpression of IL13Rα2 in human colon cancer samples was associated to metastasis and poor outcome of patients [4].

Because of the short cytoplasmic tail of IL13Rα2 (14 amino acids), it was initially regarded as a decoy, unable to transduce cell signaling. Recently, several articles described that the binding of IL13 to IL13Rα2 triggered the activation of several proteins, such as Src, AKT and AP-1 [4, 5]. By a proteomic approach using colon cancer cells, we identified the proteins that co-immunoprecipitated with IL13Rα2 and found a scaffold protein, FAM120A, also known as C9orf10 or OSSA [6], as a major interactor. FAM120A was overexpressed in colorectal cancer cell lines and 55% of human colon cancer specimens. Still, FAM120A was a quite uncharacterized protein, with no catalytic activity, which was described either as a promoter of Src activation by oxidative stress or a component of RNA-containing structures. Therefore, we investigated the protein network of FAM120A by quantitative proteomics. Proteins associated with FAM120A were involved in three cell membrane complexes: TRAIL receptor, G protein coupled receptor (GPR56) and matrix-cell adhesion networks. Many of the proteins belonging to these complexes were signaling proteins, suggesting that FAM120A facilitates the interaction and activation of such proteins after different extracellular stimuli. In fact, IL13 triggered the recruitment into the IL13Rα2/FAM120A complex and the activation of several signaling molecules, such as FAK, Src, PI3K, AKT, mTOR and ERK1/2. FAM120A was required for the activation of PI3K/AKT/mTOR pathway by Src kinases and for the activation of FAK.

Moreover, we found that FAM120A was associated to proteins involved in vesicle trafficking, suggesting a role in protein transport that is common in scaffold proteins. In fact, the silencing of FAM120A provoked a partial reduction in IL13Rα2 expression in the cell membrane, whereas total IL13Rα2 expression remained unchanged. Moreover, FAM120A silencing caused a significant downregulation of other scaffold proteins and linkers to cytoskeleton, such as AHNAK, SH3PXD2B, SPTAN1, PDLIM7 and DSG2 as well as some proteins involved in protein transport such as MYO1D, S100A10 and ACTN4. Finally, FAM120A silencing down-regulated three proteins involved in RNA splicing (NUDT21, SFPQ and SF382), which may explain the previous link between FAM120A and RNA biology.

In summary, FAM120A is a scaffold protein required for the proper IL13Rα2-triggered signaling, which is involved in colon cancer metastasis. FAM120A also modulates IL13Rα2 cell membrane location and promotes the expression of other scaffold proteins involved in signaling or transport. These results support the use of FAM120A as potential target for therapy and reinforce the therapeutic value of IL13Rα2 in colon cancer. In other cancer types, where IL13Rα2 has been described as a poor prognosis biomarker, FAM120A could also play an important role as regulator of IL13-triggered signaling. In fact, according to cBioPortal [7], FAM120A is overexpressed in sarcoma (28%), head and neck squamous cell carcinoma (24%), adrenocortical carcinoma (20%), pancreatic adenocarcinoma (12%) and brain glioma (12%). Furthermore, a role for IL13Rα2 has been shown for diverse pathologies, as asthma or ulcerative colitis. FAM120A is associated to other complexes and may play a role in the signaling after other stimuli besides IL13. We found FAM120A related with focal adhesion proteins where may collaborate in FAK activation. Also, FAM120A may be involved in GPR56 signal transduction, as this receptor for type III collagen activates RhoA, and FAM120A is associated to RhoGEFs. Finally, in some cancer cell types proapoptotic TRAIL receptor is actually promoting survival by activation of different kinases, including PI3K. FAM120A is associated to a TRAIL receptor and promotes PI3K activation; thus, it may have a potential role in anti-apoptotic conversion of the TRAIL receptor. Therefore, the role of IL13Rα2/FAM120A in different pathologies open new research lines with important implications in asthma or ulcerative colitis.
